# Longitudinal assessment of demographic representativeness in the Medical Imaging and Data Resource Center open data commons

**DOI:** 10.1117/1.JMI.10.6.061105

**Published:** 2023-07-18

**Authors:** Heather M. Whitney, Natalie Baughan, Kyle J. Myers, Karen Drukker, Judy Gichoya, Brad Bower, Weijie Chen, Nicholas Gruszauskas, Jayashree Kalpathy-Cramer, Sanmi Koyejo, Rui C. Sá, Berkman Sahiner, Zi Zhang, Maryellen L. Giger

**Affiliations:** a University of Chicago, Chicago, Illinois, United States; b The Medical Imaging and Data Resource Center (midrc.org); c Puente Solutions LLC, Phoenix, Arizona, United States; d Emory University, Atlanta, Georgia, United States; e National Institutes of Health, Bethesda, Maryland, United States; f United States Food and Drug Administration, Silver Spring, Maryland, United States; g University of Colorado Anschutz Medical Campus, Aurora, Colorado, United States; h Stanford University, Stanford, California, United States; i University of California, San Diego, La Jolla, California, United States; j Jefferson Health, Philadelphia, Pennsylvania, United States

**Keywords:** COVID-19, representativeness, fairness, artificial intelligence, medical imaging

## Abstract

**Purpose:**

The Medical Imaging and Data Resource Center (MIDRC) open data commons was launched to accelerate the development of artificial intelligence (AI) algorithms to help address the COVID-19 pandemic. The purpose of this study was to quantify longitudinal representativeness of the demographic characteristics of the primary MIDRC dataset compared to the United States general population (US Census) and COVID-19 positive case counts from the Centers for Disease Control and Prevention (CDC).

**Approach:**

The Jensen-Shannon distance (JSD), a measure of similarity of two distributions, was used to longitudinally measure the representativeness of the distribution of (1) all unique patients in the MIDRC data to the 2020 US Census and (2) all unique COVID-19 positive patients in the MIDRC data to the case counts reported by the CDC. The distributions were evaluated in the demographic categories of age at index, sex, race, ethnicity, and the combination of race and ethnicity.

**Results:**

Representativeness of the MIDRC data by ethnicity and the combination of race and ethnicity was impacted by the percentage of CDC case counts for which this was not reported. The distributions by sex and race have retained their level of representativeness over time.

**Conclusion:**

The representativeness of the open medical imaging datasets in the curated public data commons at MIDRC has evolved over time as the number of contributing institutions and overall number of subjects have grown. The use of metrics, such as the JSD support measurement of representativeness, is one step needed for fair and generalizable AI algorithm development.

## 1 Introduction

Since the first identification of the SARS-CoV-2 coronavirus (and its associated infectious disease, COVID-19) in late 2019, there have been reports of differences in disease health outcomes by race, ethnicity, sex, and other demographics.^1^^–^^14^ Additionally, the relative difference in impact of COVID-19 to demographic subgroups has also changed over time.^15^^,^^16^ Furthermore, differences in the utilization of medical imaging in healthcare have been observed in various demographic subgroups over the course of the pandemic.^17^^–^^19^ As a result, the use of medical imaging among different demographic subpopulations can be expected to change over time.

The Medical Imaging and Data Resource Center (MIDRC)^20^ is a multi-institutional initiative designed to collect, curate, and share medical images and other related resources to support the development of artificial intelligence/machine learning (AI/ML) for diagnosis, treatment, and prognosis of COVID-19 and beyond. MIDRC is hosted at the University of Chicago, funded by the National Institute of Biomedical Imaging and Bioengineering, and co-led by the American College of Radiology^®^ (ACR), the Radiological Society of North America (RSNA), and the American Association of Physicists in Medicine (AAPM). Studies are contributed to MIDRC by institutions via a pipeline that includes a collaborative partnership between the ACR^®^, the RSNA, the AAPM and Gen 3, a data commons organization. Users can access the data under either a non-commercial research or a commercial use agreement.^21^ MIDRC places a strong emphasis on monitoring and increasing the representativeness of the data, both at specific instances in time and longitudinally, to help support the development of unbiased and generalizable algorithms.

The purpose of this study was to (1) introduce the use of a metric to measure representativeness of the imaging datasets compared to relevant groups and (2) report on the evolution of the representativeness since the ingestion of datasets from contributors began in August 2021.

## 2 Materials and Methods

### 2.1 Dataset

Data used in this study were composed of metadata for the imaging studies available at the MIDRC open data commons^22^ in the open-A1 and open-R1 datasets (i.e., those ingested by the ACR^®^ and RSNA and curated and harmonized by AAPM and Gen3). In this study, we refer to this specific collection as “MIDRC data.” The metadata had been submitted by data contributors in accordance with the MIDRC data dictionary.^23^ Assignment of unique patients into the open data commons occurs at the ingestion of data within MIDRC according to a multidimensional stratified sampling algorithm,^24^ with ∼80% of unique patients assigned to the open data commons and 20% of unique patients assigned to a sequestered data commons. The sequestration algorithm is designed and tested for balance among groupings including but not limited to demographic categories.

For the purposes of this study, demographic categories were analyzed as follows: age at index event (i.e., the first occurrence in MIDRC, usually the first COVID-19 test), sex, race, ethnicity, and the combination of race and ethnicity. The latter was used in accordance with guidance from the Office of Management and Budget, which identifies patients as Hispanic or, if non-Hispanic, by their race.^25^ Patients may have multiple imaging studies in MIDRC but, for each unique patient, the characteristics at the index event were used in this study.

### 2.2 Comparison Groups

The demographic distributions of unique patients in the MIDRC data were compared against two relevant population distributions. Because at the time of this study all open-A1 and open-R1 data contributing to the MIDRC data described here had been collected in the United States, we compared the demographic distributions (1) between all cases (unique patients) within the MIDRC data and population in the United States 2020 Census^26^ and (2) between COVID-19 positive cases within the MIDRC data and population in the COVID-19 case surveillance public use data from the Centers for Disease Control and Prevention (CDC).^27^

### 2.3 Statistical Analysis

The Jensen-Shannon distance^28^^–^^30^ (JSD) was used as a metric to measure the difference between any two population categorical distributions, with the two comparison groups being termed S and T in this study. It is based upon the Jensen-Shannon divergence^31^ (called $D_{\mathrm{JS}}$ in this study) and the Kullback-Leiber divergence $D_{\mathrm{KL}}$. The $D_{\mathrm{KL}}$ is defined for two distributions as

$$D_{\mathrm{KL}}(S∥T)=\sum_{x}S(x)\log_{2}\;\frac{S(x)}{T(x)},$$ (1)

where S(x) and T(x) are the distribution functions of any two populations S and T, and x is the variable of interest which in this study is any of the demographic variables under investigation. Because all the demographic variables x in this study are discrete, the distribution functions a probability mass functions (i.e., represented by the fraction of patients at each bin of x). Subsequently, the JSD is defined as

$$\mathrm{JSD}=\sqrt{D_{\mathrm{JS}}(S∥T)},$$ (2)

where

$$D_{\mathrm{JS}}(S∥T)=\frac{1}{2}D_{\mathrm{KL}}(S∥M)+\frac{1}{2}D_{\mathrm{KL}}(T∥M)\;,$$ (3)

and

$$M=\frac{1}{2}(S+T).$$ (4)

The logarithm within $D_{\mathrm{KL}}$ can be determined through other bases (such as the natural logarithm). When $\log_{2}$ is used, the $D_{\mathrm{JS}}$ and JSD are bounded between 0 and 1, which is advantageous for our purpose. The sum in equation (1) was taken over each bin x for which both comparison groups were non-zero. A JSD of zero indicates that there is no difference between compared distributions, while a JSD of 1 indicates that there is no similarity between them. In this study, more representative distributions (compared to the reference distribution) will have a lower JSD.

In this study, the JSD was used to compare the following distributions at each MIDRC batch ingestion date:

1. cumulative counts of all unique patients in the MIDRC data to the US Census counts (${\mathrm{JSD}}_{\mathrm{MIDRC}\;(\text{all})\;\text{to census}}$),
2. the cumulative counts of all unique COVID-19 positive cases in the MIDRC data to the cumulative COVID-19 positive counts (derived from case counts) reported by the CDC (${\mathrm{JSD}}_{\mathrm{MIDRC}\;(C19+)\;\text{to}\;\mathrm{CDC}\;(C19+)}$), and
3. the cumulative COVID-19 positive counts reported by the CDC to the US Census counts (${\mathrm{JSD}}_{\mathrm{CDC}\;(C19+)\;\text{to census}}$).

The CDC to US Census comparison was used as a reference against which the comparison of MIDRC distributions can be considered.

Additionally, the temporal difference in the JSD was determined between the JSD when comparing all cases in MIDRC to the US Census and the JSD when comparing all COVID-19 positive cases to the COVID-19 positive case counts from the CDC. If this difference is positive, the distribution of COVID-19 positive unique patients in MIDRC to the CDC cumulative COVID-19 case counts is more representative than the distribution of all unique patients in MIDRC to the US general population. If this difference is negative, the distribution of all unique patients in MIDRC to the US general population is more representative than the distribution of COVID-19 positive unique patients in MIDRC to the CDC cumulative case counts.

Note that in this study, no measures of statistical difference were assessed, since the goal of the measure of representativeness here is to measure degree of similarity according to counts. Additionally, no sampling of distributions was conducted, due to the nature of the data (counts of individuals), none of which are inherently considered samples in this study.

## 3 Results

### 3.1 Dataset

**Fig. 1:**
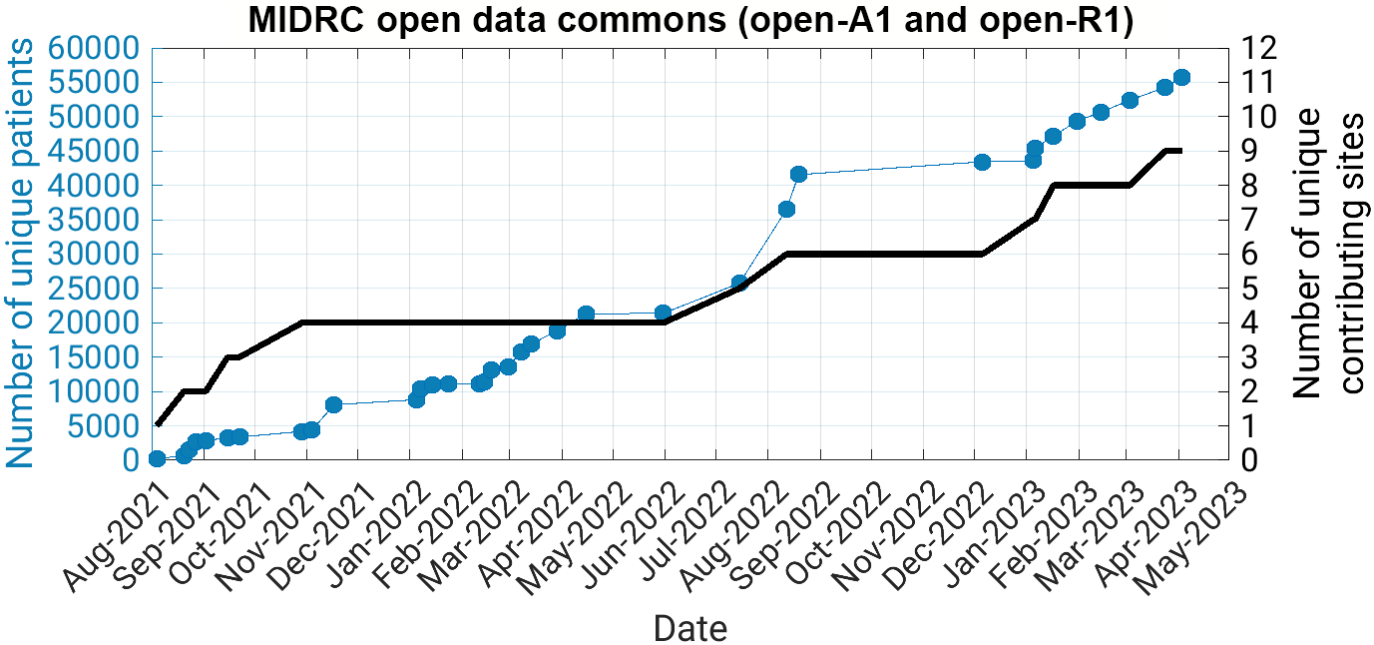
Cumulative number of unique patients and number of unique contributing sites in the open MIDRC data commons since the launch of the data commons in August 2021 through time of manuscript preparation.

As of April 3, 2023 (the most recent batch ingestion date at time of manuscript preparation), there were 9 unique contributing sites and over 55,000 unique patients represented in the MIDRC data (Fig. 1).

**Fig. 2:**
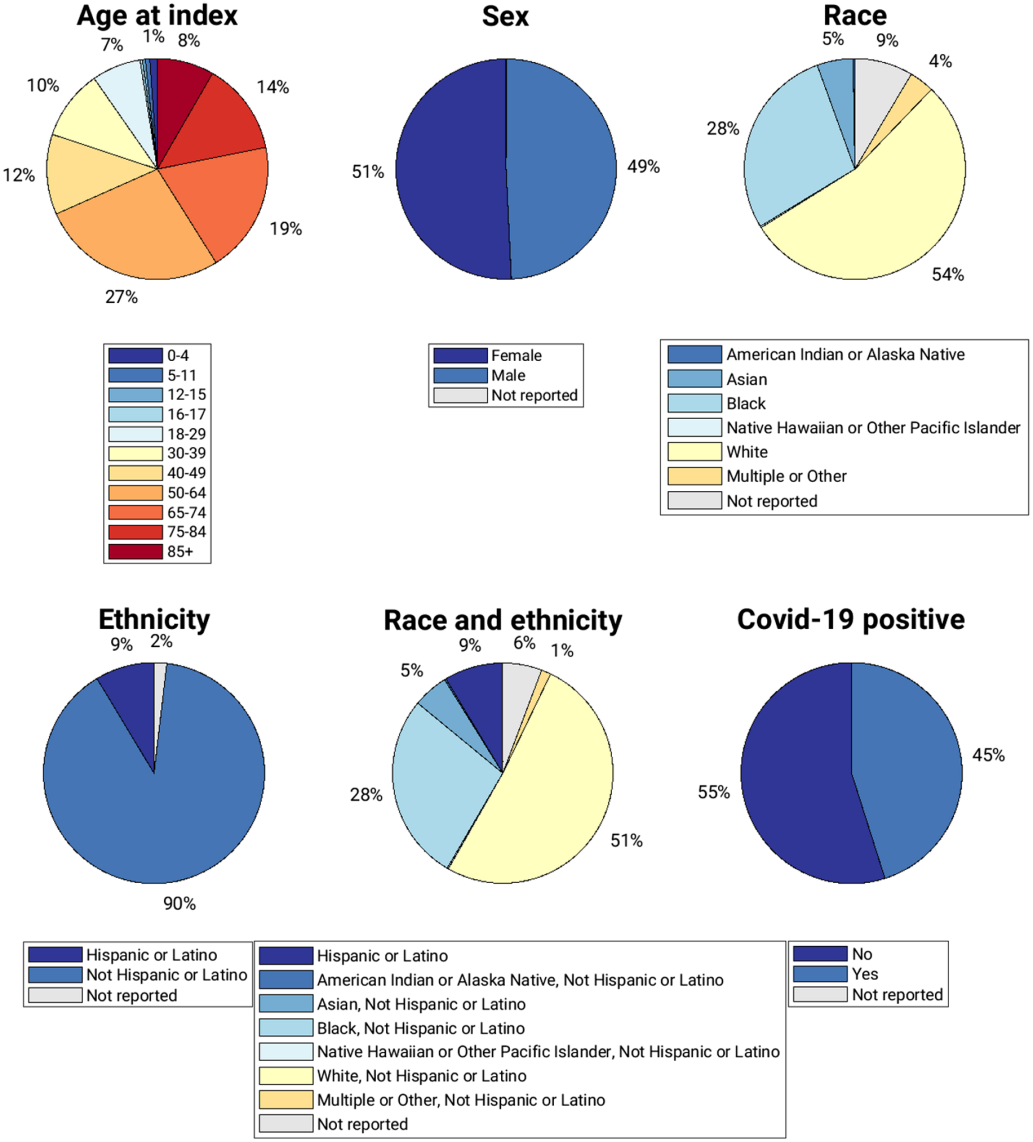
Pie charts of the percentages of unique patients in the MIDRC data as of April 3, 2023 by demographic category and COVID-19 status. The presentation of demographic data in pie chart form here is the same as the bar graph representation for “MIDRC (all)” (blue bars) in each subfigure of Fig. 3.

The proportions of the MIDRC data by demographic category and COVID-19 status as of April 3, 2023 are given in Fig. 2.

**Fig. 3:**
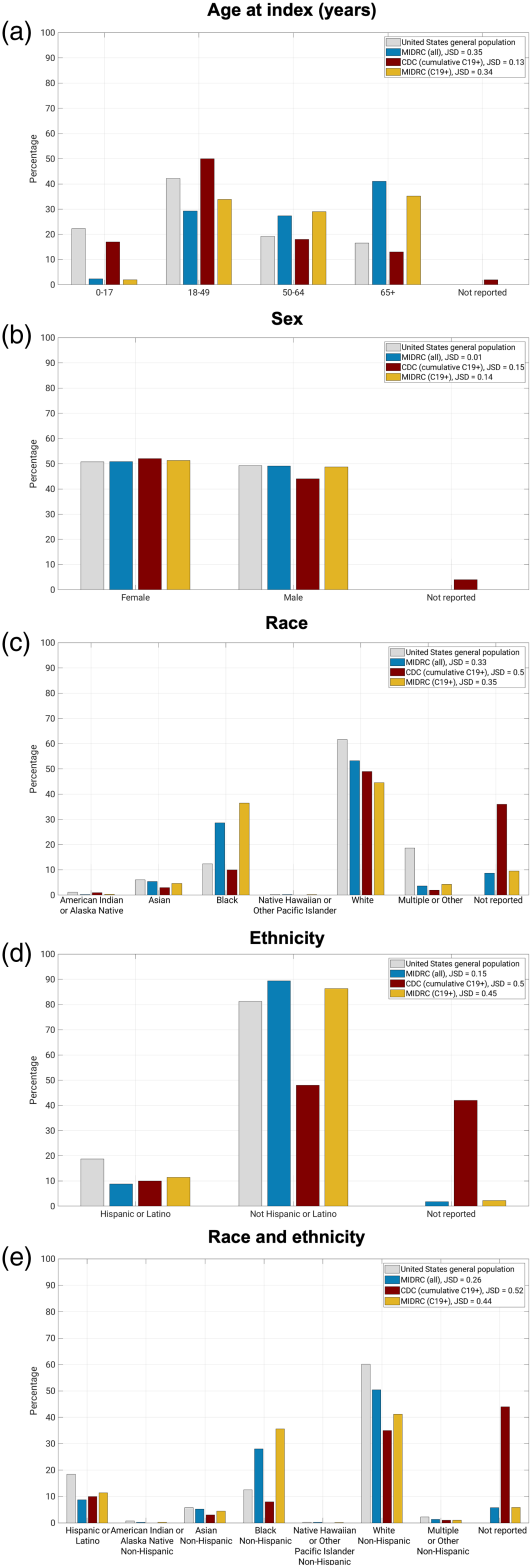
Distributions of cases within the MIDRC data as of March 24, 2023 (the latest ingestion date that can be compared to CDC data) and comparison groups (US general population from the 2020 census and cumulative case counts from the CDC). In these figures, the JSD is shown for (1) all cases in the MIDRC data compared to the US general population (${\mathrm{JSD}}_{\mathrm{MIDRC}\;(\text{all})\;\text{to census}}$), (2) cumulative CDC COVID-19 positive case counts compared to the US general population (${\mathrm{JSD}}_{\mathrm{CDC}\;(C19+)\;\text{to census}}$), and (3) MIDRC COVID-19 positive case counts compared to the CDC COVID-19 positive case counts (${\mathrm{JSD}}_{\mathrm{MIDRC}\;(C19+)\;\text{to}\;\mathrm{CDC}\;(C19+)}$). The JSD is bounded between 0 and 1, where 0 indicates that two distributions are the same as measured by the JSD and 1 indicates that they are completely different. MIDRC, The Medical Imaging and Data Resource Center; US, United States; CDC, Centers for Disease Control and Prevention; and C19+, cases positive for COVID-19. (a) Age at index; (b) sex; (c) race; (d) ethnicity; (e) race and ethnicity.

The most recent distributions of unique patients within each demographic category, both within the MIDRC data and the comparison groups, are given in Fig. 3 along with JSD results. Longitudinal measurements of the demographic data for the MIDRC data are available in the Supplementary Material.

**Fig. 4:**
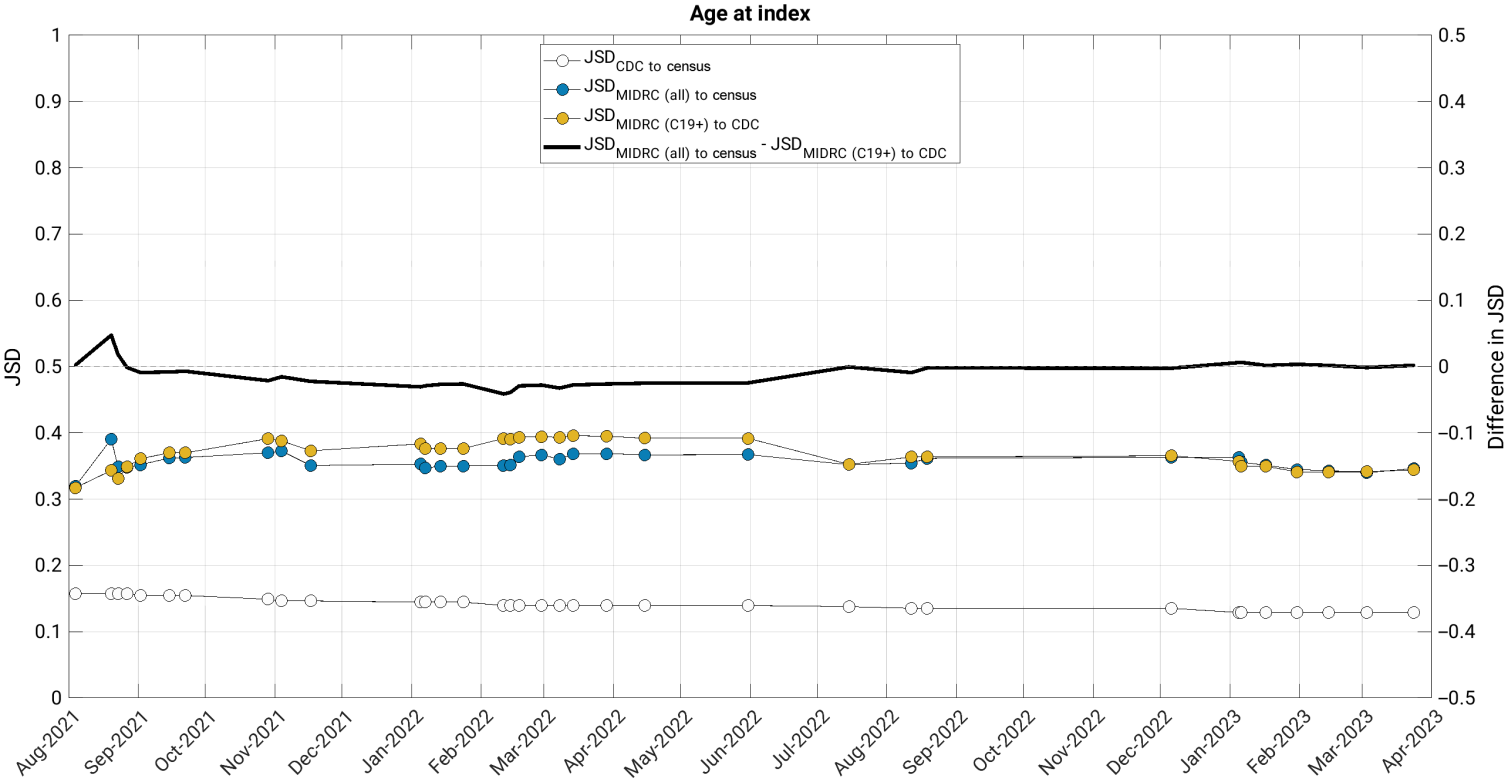
The Jensen-Shannon distance (JSD) over time for age at index for (blue data markers) all unique patients, (gold data markers) all unique COVID-19 positive patients in the MIDRC data, and (white data markers) the JSD for comparing the CDC data to the US general population (for reference). The difference in JSD over time between all unique patients and all unique COVID-19 positive patients in the MIDRC data is also shown (black line). The similarity of both all unique patients and all unique COVID-19 positive patients in the MIDRC data has remained fairly constant to their respective comparison groups over time in terms of JSD. MIDRC, The Medical Imaging and Data Resource Center; US, United States; CDC, Centers for Disease Control and Prevention; and C19+, cases positive for COVID-19.

**Fig. 5:**
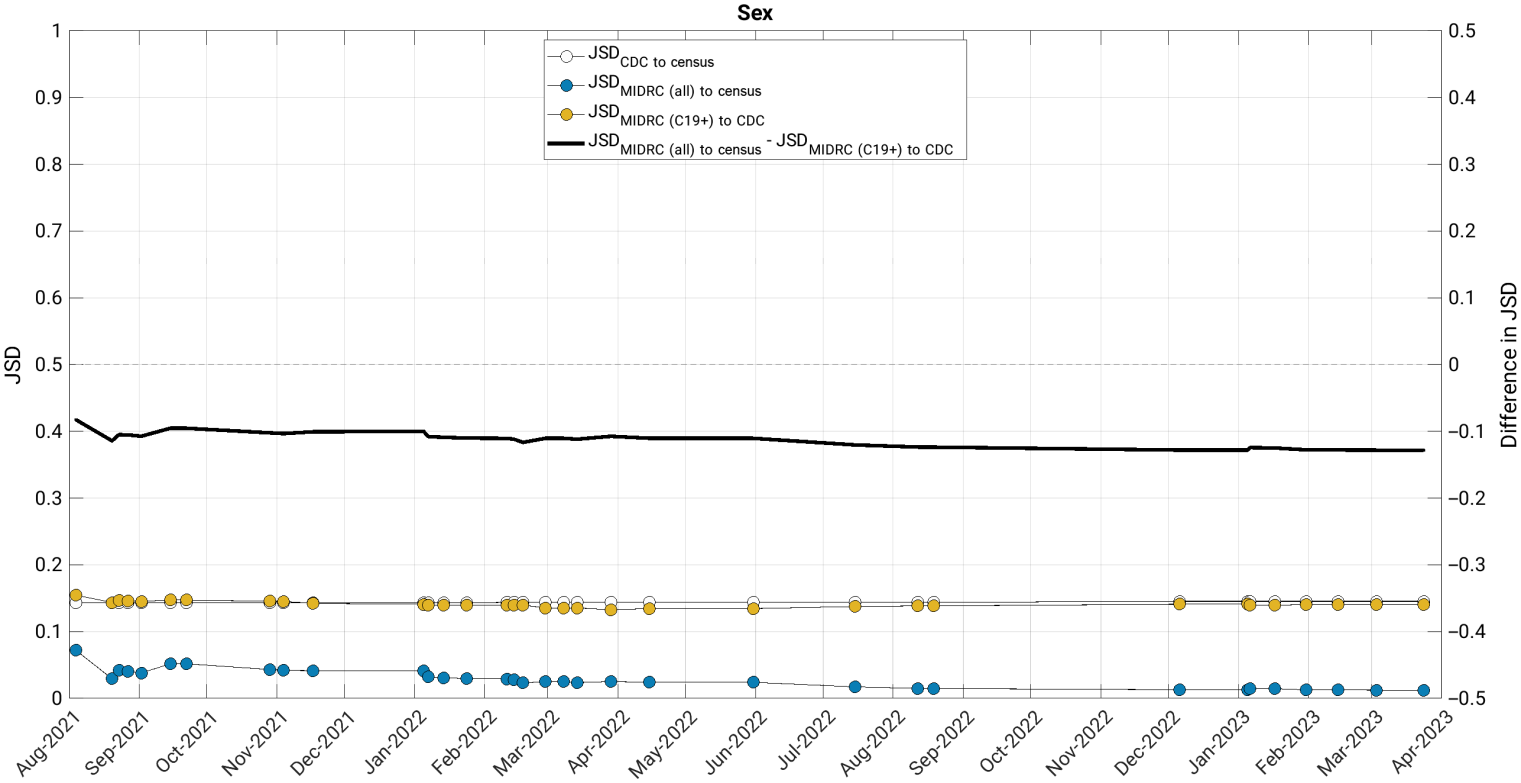
The Jensen-Shannon distance (JSD) over time for sex for (blue data markers) all unique patients, (gold data markers) all unique COVID-19 positive patients in the MIDRC data, and (white data markers) the JSD for comparing the CDC data to the US general population (for reference). The difference in JSD over time between all unique patients and all unique COVID-19 positive patients in the MIDRC data is also shown (black line). The distribution of all unique cases in the MIDRC data has been more representative of the US population than the distribution of all unique COVID-19 cases to the CDC cumulative case counts, and this higher representativeness has slightly increased as the number of unique cases has increased. MIDRC, The Medical Imaging and Data Resource Center; US, United States; CDC, Centers for Disease Control and Prevention; and C19+, cases positive for COVID-19.

**Fig. 6:**
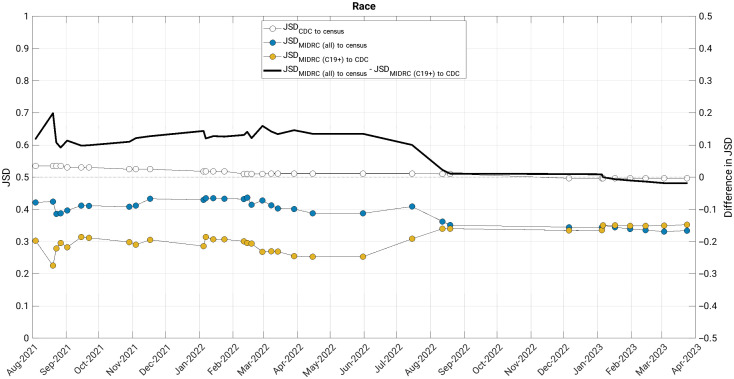
The Jensen-Shannon distance (JSD) over time for race for (blue data markers) all unique patients, (gold data markers) all unique COVID-19 positive patients in the MIDRC data, and (white data markers) the JSD for comparing the CDC data to the US general population (for reference). The difference in JSD over time between all unique patients and all unique COVID-19 positive patients in the MIDRC data is also shown (black line). The distribution of unique patients in the MIDRC data has recently reached similar levels of representativeness to their comparison groups (difference in JSD approaching zero). MIDRC, The Medical Imaging and Data Resource Center; US, United States; CDC, Centers for Disease Control and Prevention; and C19+, cases positive for COVID-19.

**Fig. 7:**
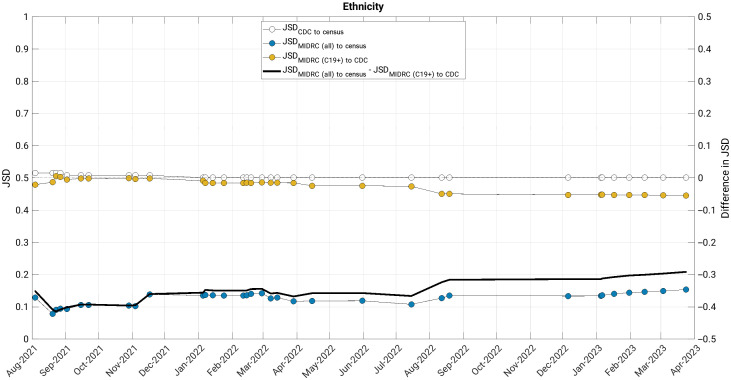
The Jensen-Shannon distance (JSD) over time for ethnicity for (blue data markers) all unique patients, (gold data markers) all unique COVID-19 positive patients in the MIDRC data, and (white data markers) the JSD for comparing the CDC data to the US general population (for reference). The difference in JSD over time between all unique patients and all unique COVID-19 positive patients in the MIDRC data is also shown (black line). The distribution comparisons are substantially similar for all unique patients in MIDRC to the United States general population than all unique COVID-19 positive patients to the case count distributions from the Centers for Disease Control and Prevention (CDC) due to the high percentage of cases within the CDC data for which ethnicity is not available (over 40%). MIDRC, The Medical Imaging and Data Resource Center; US, United States; CDC, Centers for Disease Control and Prevention; and C19+, cases positive for COVID-19.

**Fig. 8:**
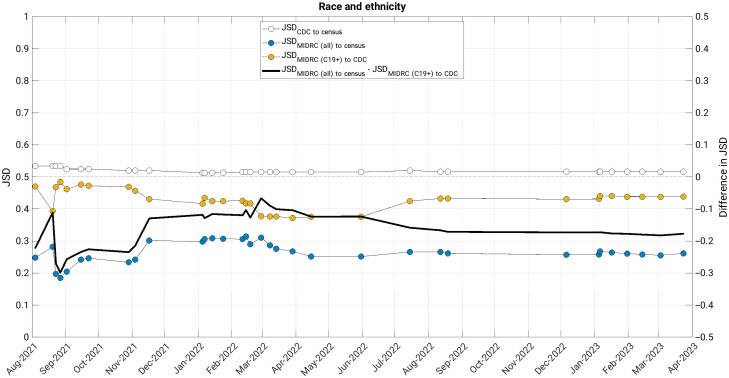
The Jensen-Shannon distance (JSD) over time for race and ethnicity for (blue data markers) all unique patients, (gold data markers) all unique COVID-19 positive patients in the MIDRC data, and (white data markers) the JSD for comparing the CDC data to the US general population (for reference). The difference in JSD over time between all unique patients and all unique COVID-19 positive patients in the MIDRC data is also shown (black line). The lower representativeness (higher JSD) of all unique COVID-19 positive patients in the MIDRC data to the CDC data and the CDC data to the US general population is impacted by the substantial percentage of cases within the data from the Centers for Disease Control and Prevention for which race and ethnicity is not reported (over 40%). MIDRC, The Medical Imaging and Data Resource Center; US, United States; CDC, Centers for Disease Control and Prevention; and C19+, cases positive for COVID-19.

The JSD measured changes in the similarity of the MIDRC data (both when considering all imaging studies and those from COVID-19 positive patients only) to their comparison groups (the United States general population and case counts from the CDC, respectively) (Figs. 4–8). The comparison of age at index to the United States general population and the cumulative case counts as recorded by the CDC has remained relatively stable in these sets of patients over time, with little difference in their level of representativeness (Fig. 4).

The comparison of MIDRC unique patients by sex to the United States general population and the cumulative case counts as recorded by the CDC has demonstrated more representativeness (i.e., more similarity) in the distribution of all unique patients to the United States general population (lower JSD) than the comparison of MIDRC positive patients to the cumulative case count from the CDC (higher JSD) (Fig. 5). Over time, there has been a slight increase in the difference of the comparisons as the proportion of male unique patients has increased.

The representativeness of MIDRC unique patients by race to the United States general population and the cumulative case counts as recorded by the CDC reached almost equal similarity within the MIDRC data in August 2022 (Fig. 6). However, it is important to note that the measurement of representativeness for all three comparisons is impacted by the proportion of subjects for which race is not reported, which is over 30% in the most recent CDC cumulative case counts and around 10% in the most recent distributions within the MIDRC data. The MIDRC data also have substantially higher proportions of unique patients with reported race as Black than the US general population and the CDC cumulative case counts.

The comparison of MIDRC unique patients by ethnicity to the United States general population has been substantially more similar than the comparison of MIDRC COVID-19 positive patients to the cumulative CDC case counts (Fig. 7). This is likely a result of the substantial percentage of cases within the CDC data for which ethnicity is not available (over 40%).

The comparison of MIDRC unique patients by the combination of race and ethnicity to the United States general population has been more similar than the comparison of MIDRC COVID-19 positive patients to the cumulative CDC case counts (Fig. 8). This may also be impacted by the substantial percentage of cases within the CDC data for which race and ethnicity is not available.

## 4 Discussion

The representativeness of the MIDRC data continues to change over time as the number of contributing institutions and the overall number of unique patients grow. The evolution of the impact of the COVID-19 pandemic to various demographic groups also continues to change over time, as shown by the changes in ${\mathrm{JSD}}_{\mathrm{CDC}\;(C19+)\;\text{to census}}$ (not discussed in detail here). Using the JSD contributes to quantifying the comprehensive representativeness of the data and supports several initiatives related to health-related research and development at the federal level, including the strategic plan of the National Institute on Minority Health and Health Disparities^32^ (specifically, Goal 7: “ensure appropriate representation of minority and other health disparity populations in NIH-funded research”) and action plans and guidance from the Food and Drug Administration.^33^^–^^35^

The goal of the development of fair and generalizable AI/ML algorithms in medical imaging has rightfully been the topic of much attention.^36^^–^^43^ Goals of algorithmic fairness and generalizability involve those of equal outcomes (including equalized outcomes) and of equal performance.^44^ It should be noted that defining the representativeness of data is a crucial part of developing and deploying algorithms with fairness and generalizability in mind. Indeed, defining representativeness is one of many careful procedures needed in AI/ML, with others including but not limited to definition of the purpose of data collection, aim of the model, and careful identification of the task. As has been noted by others,^45^ representativeness can involve representativity of data in the sense of coverage of the “input space” (i.e., the training and/or the test data) and/or representativeness to population distributions. The use of the JSD generally can support the assessment of representativeness of distributions for either aim; in this study, we used the JSD to measure representativeness of the MIDRC data to demographic distributions.

Assessment of representativeness of data by demographic categories is but one part of ensuring fairness and generalizability at various stages of AI/ML pipelines. These include data collection (by identifying protected groups and their representation and addressing unequal representation in data through intentional collection efforts), model tuning and evaluation (such as comparing deployment data with training data across subgroups), and performance monitoring (including monitoring for data shifts, such as changes of impact in disease to subgroups over time^44^^,^^46^). Population characterization (and potentially matching synthetically^47^^,^^48^), cross-population modeling,^49^^,^^50^ and class balancing^51^ can be useful in AI/ML algorithm development to identify and avoid model bias.

We believe the Jensen-Shannon distance to be useful in AI/ML investigations in medical imaging, in part due to its intuitive nature (especially in terms of its bounds) and its relationship to information theory, which is an important foundation to other AI/ML performance measures such as receiver operating characteristic analysis.^52^ Jensen-Shannon measures have also been used in some biology studies.^53^ There are other methods for comparing the distributions of populations, such as the Hellinger distance,^54^ population matching discrepancy,^55^ and matching quantiles estimation.^56^ The ratio of patient identity to disease prevalence, termed the participant to prevalence ratio (PPR),^57^ is used in some clinical trials (in which subjects are termed “participants”) to measure representation within demographic subgroups. A measure such as the JSD is desirable for our definition of representativeness due to its ability to summarize across a demographic category, but measures such as the PPR would be complementary for analysis of individual subgroups. It would be advantageous for future studies to quantify the impact of ranges of PPR on adequate representation or lack thereof and to establish more specific criteria for such levels. On the whole, it will be useful to conduct a comprehensive comparison of different measures of representativeness (both across an entire demographic category and by subgroups) and their relationship to fairness of AI in medical imaging; this will be the topic of future work.

The work described here uses COVID-19 positive case counts collected by the CDC before the declared end of the COVID-19 public health emergency, on May 11, 2023. After this date, COVID-19 data reporting by the CDC will change, impacting the reporting of case counts.^58^ We will continue to monitor the representativeness of the MIDRC open data commons as batch ingestion continues, using hospitalization rates^59^ as a comparison group for COVID-19 positive cases.

There were some limitations to this study. First, the demographic categories described here were limited to one combination of demographic categories (race and ethnicity). Other combinations of demographic categories are important to consider (such as that of age and race or sex and race) and will be the topic of future study. Second, this study did not include other factors which may be relevant in studying health inequities, such as patient residence (e.g., urban versus rural), healthcare institution type (e.g., community versus academic), patient education level attainment, patient income or experienced income equities, and patient employment status. Third, there were some limitations in the data reported by the CDC: (1) it includes both probable and lab-confirmed COVID-19 cases, while COVID-19 positive cases in the MIDRC data commons include lab confirmation; (2) it includes non-unique case counts (i.e., the counts include some individuals who have tested positive for COVID-19 at different times) while the MIDRC data counts patients only once; and (3) it includes substantial proportions of data for which race and ethnicity are not reported. We are currently conducting related studies on the impact to AI/ML algorithm development and performance evaluation when representativeness is impacted by sizable proportions of missing data. While the purpose of this work is to report on the representativeness of the MIDRC open data commons *prima facie*, we also note the limitations of measures when using CDC data, including its missing data. In the future, we will investigate using methods the CDC is currently proposing to address missing data, such as assuming individuals with no reported ethnicity are non-Hispanic.^60^ Finally, MIDRC works with data contributors to receive imaging study donations that have been de-identified using the Safe Harbor method, in compliance with the Health Insurance Portability and Accountability Act of 1996 privacy rule. Thus, while each patient’s timeline is preserved, the MIDRC data commons does not provide the actual date of image acquisition and COVID test. This means that the imaging studies within a given ingestion date can include imaging studies acquired theoretically at any time before the ingestion date, necessitating our comparison of cumulative case counts in both the MIDRC data and the COVID-19 case counts from the CDC, rather than a potential comparison for cases imaged within a given month to cases reported by the CDC within a given month.

In summary, the demographic characteristics of the MIDRC data in the categories of age at imaging, sex, race, ethnicity, and the combination of race and ethnicity and their similarity to comparison groups were measured using the Jensen-Shannon distance. Overall, the JSD indicated more representativeness for all unique patients than for COVID-19 positive patients when compared to their respective comparison groups. These measures can be used by investigators in developing unbiased and generalizable AI/ML algorithms using the MIDRC data, including when building cohorts.

## Data and Materials Availability Statement

The data presented in this article are publicly available from Ref. 20.
