# Supplementary material for: Longitudinal assessment of demographic representativeness in the Medical Imaging and Data Resource Center open data commons
Source: J Med Imaging (Bellingham). 2023 Jul 18;10(6):61105. doi: 10.1117/1.JMI.10.6.061105 (PMC10353566; doi:10.1117/1.JMI.10.6.061105)
Supplement: Supplementary file 1 [file JMI_010_061105_SD001.docx]

**Supplemental data**

The longitudinal measurement of demographics within the MIDRC data has changed over time (Supplemental Figures 1-5).

| 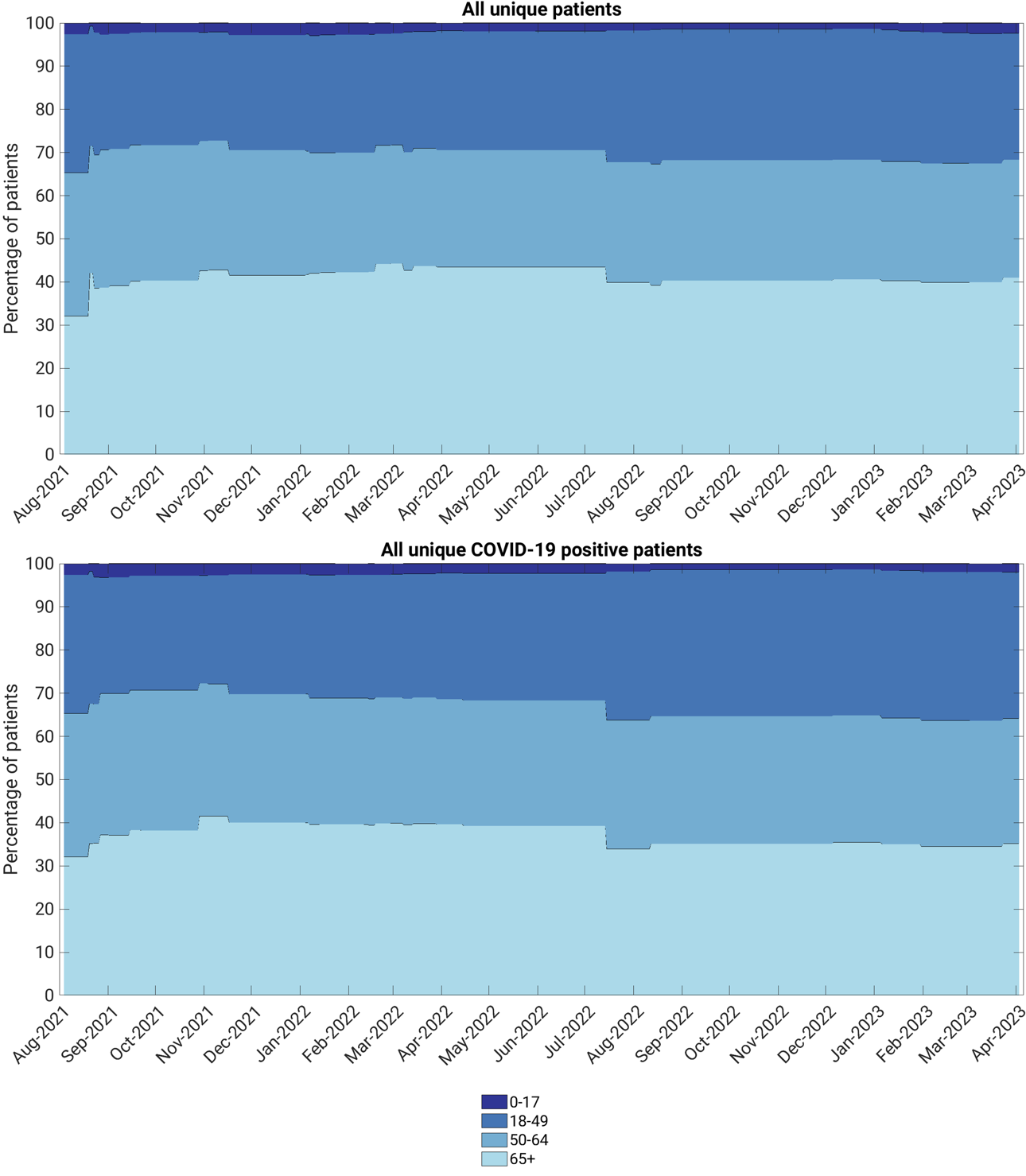 |
| --- |
| Supplemental Figure 1: Longitudinal percentage of patients in the demographic category of age (in years at index) for (top) all unique patients and (bottom) unique COVID-19 positive patients in the MIDRC data. |

| 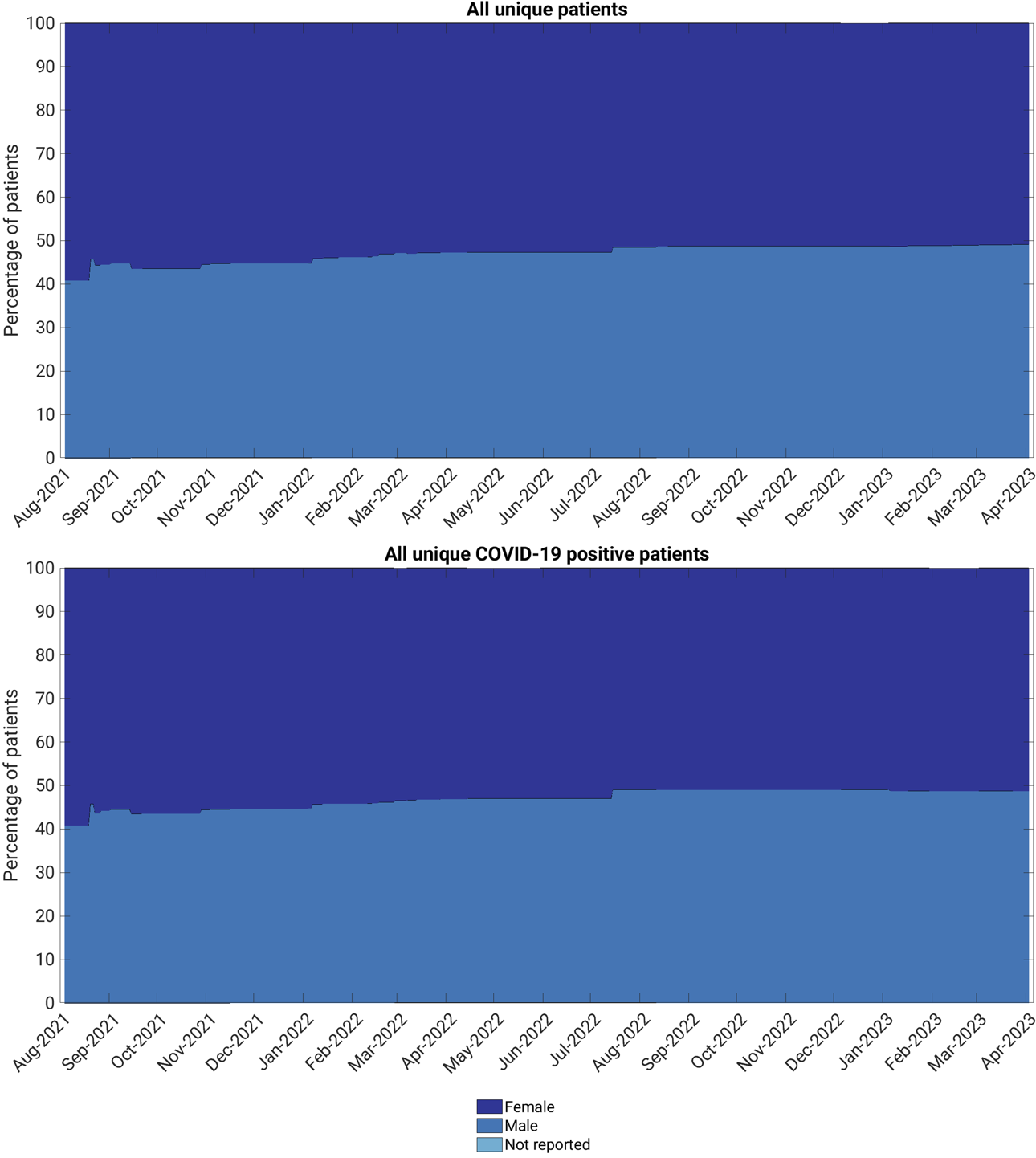 |
| --- |
| Supplemental Figure 2: Longitudinal percentage of patients in the demographic category of sex for (top) all unique patients and (bottom) unique COVID-19 positive patients in the MIDRC data. |

| 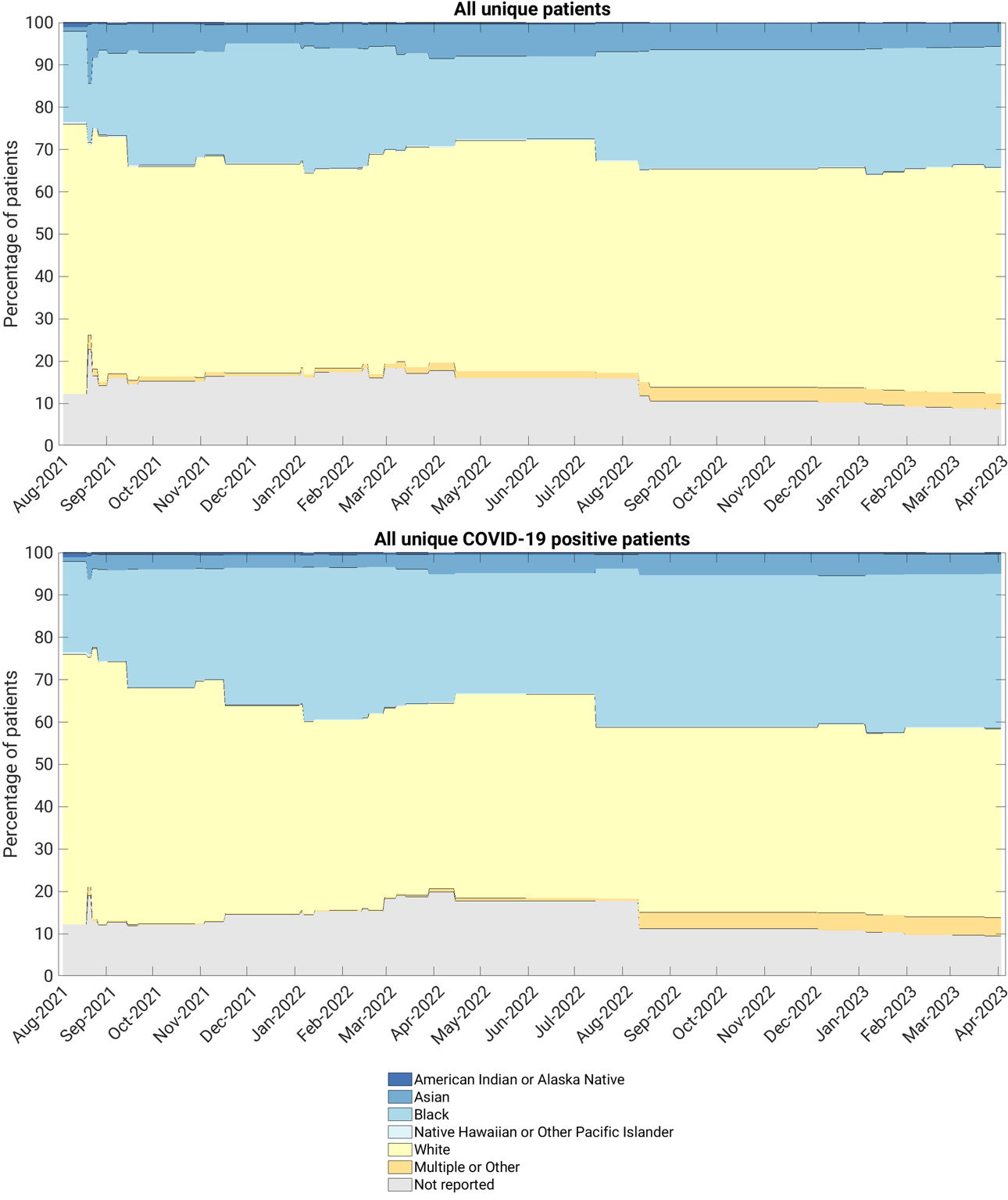 |
| --- |
| Supplemental Figure 3: Longitudinal percentage of patients in the demographic category of race for (top) all unique patients and (bottom) unique COVID-19 positive patients in the MIDRC data. |

| 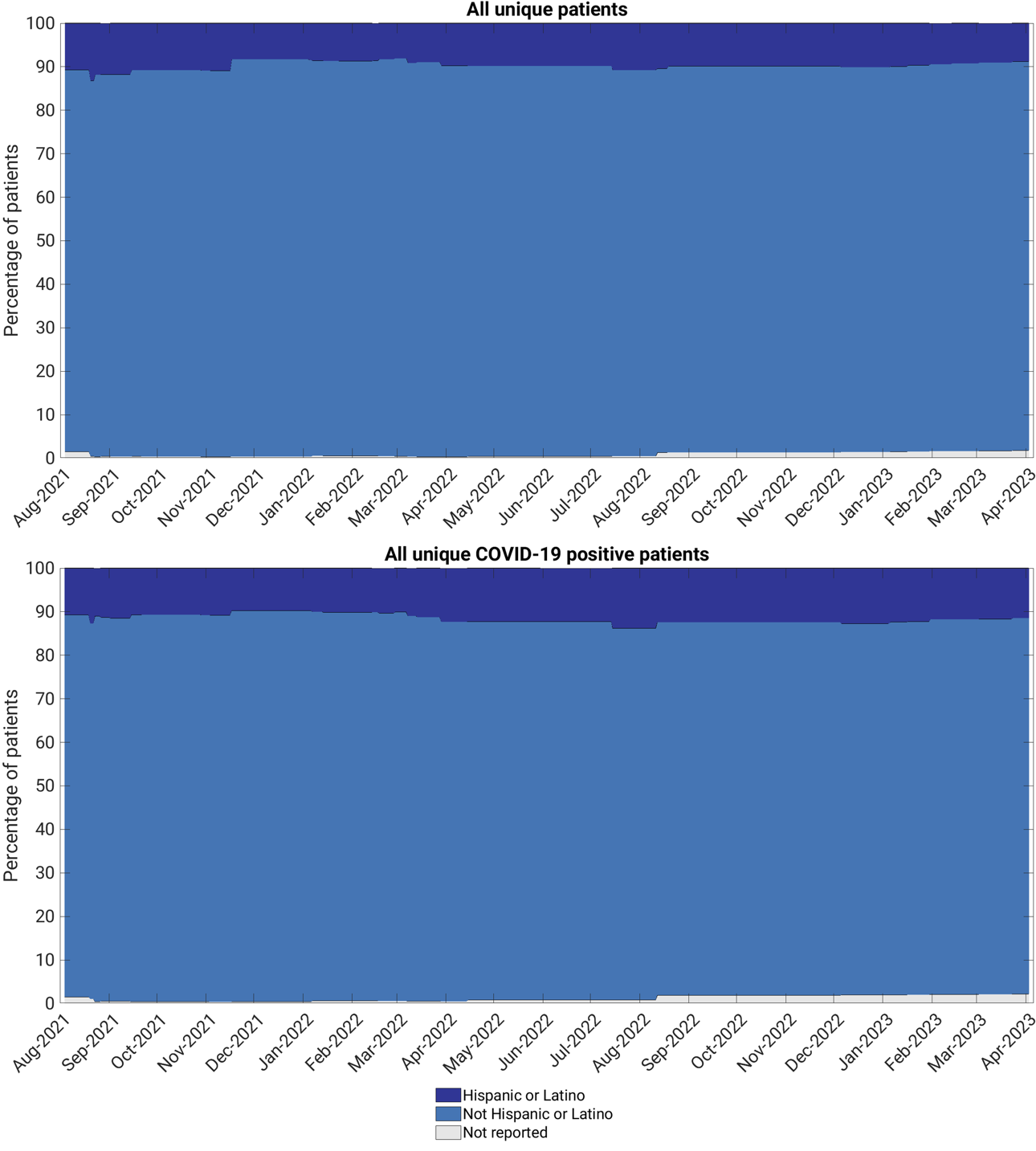 |
| --- |
| Supplemental Figure 4: Longitudinal percentage of patients in the demographic category of ethnicity for (top) all unique patients and (bottom) unique COVID-19 positive patients in the MIDRC data. |

| 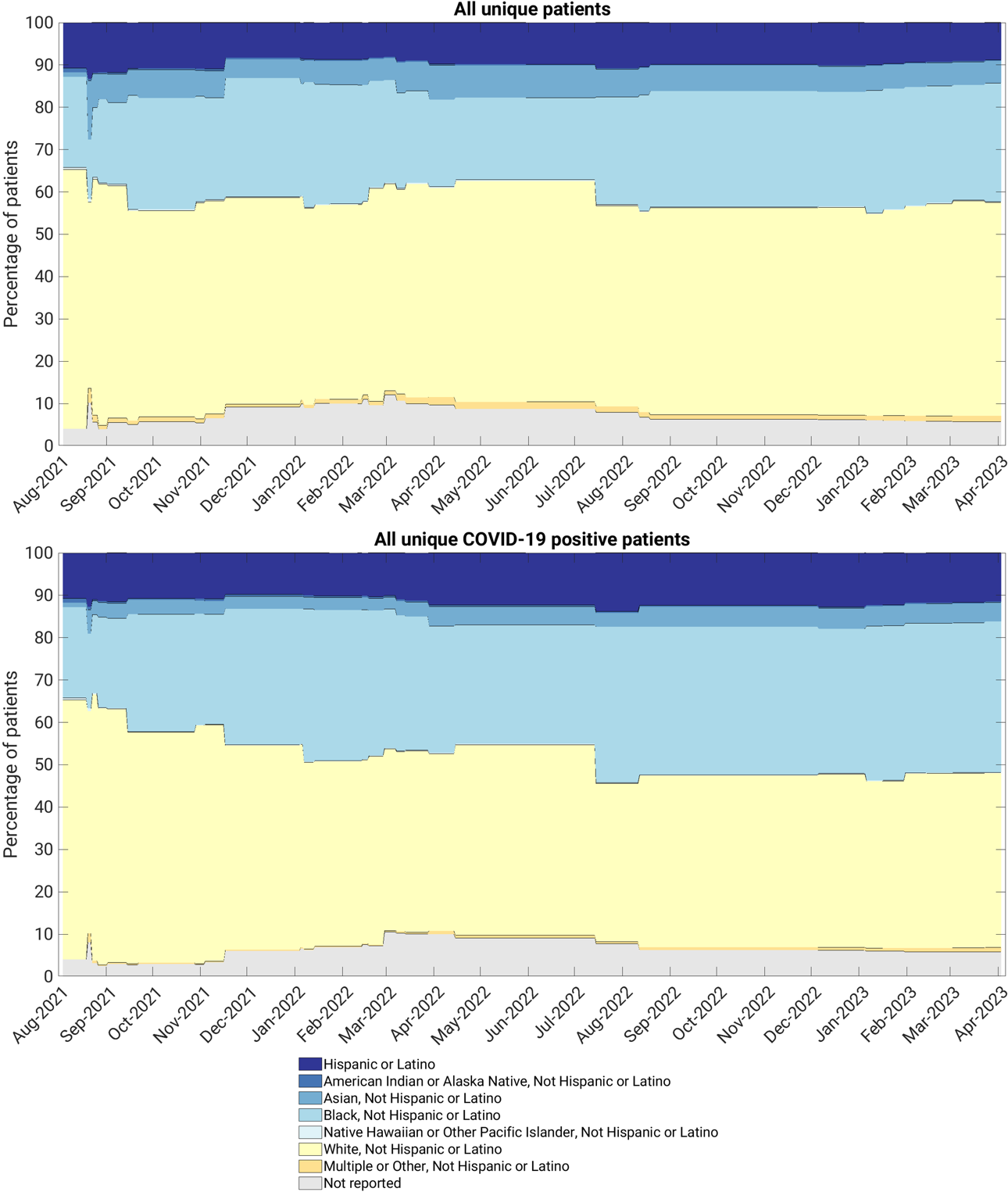 |
| --- |
| Supplemental Figure 5: Longitudinal percentage of patients in the demographic category of combination of race and ethnicity for (top) all unique patients and (bottom) unique COVID-19 positive patients in the MIDRC data. |
